# Design and Characterization of an Ethosomal Gel Encapsulating Rosehip Extract

**DOI:** 10.3390/gels9050362

**Published:** 2023-04-25

**Authors:** Valentina Sallustio, Giovanna Farruggia, Massimiliano Pio di Cagno, Martina M. Tzanova, Joana Marto, Helena Ribeiro, Lidia Maria Goncalves, Manuela Mandrone, Ilaria Chiocchio, Teresa Cerchiara, Angela Abruzzo, Federica Bigucci, Barbara Luppi

**Affiliations:** 1 Drug Delivery Research Laboratory, Department of Pharmacy and Biotechnology, Alma Mater Studiorum, University of Bologna, Via San Donato 19/2, 40127 Bologna, Italy; valentina.sallustio2@unibo.it (V.S.); angela.abruzzo2@unibo.it (A.A.); federica.bigucci@unibo.it (F.B.); barbara.luppi@unibo.it (B.L.); 2 Pharmaceutical Biochemistry Laboratory, Department of Pharmacy and Biotechnology, Alma Mater Studiorum, University of Bologna, Via San Donato 19/2, 40127 Bologna, Italy; giovanna.farruggia@unibo.it; 3 Department of Pharmacy, Faculty of Mathematics and Natural Sciences, University of Oslo, Sem Saelands vei 3, 0371 Oslo, Norway; m.p.d.cagno@farmasi.uio.no (M.P.d.C.); m.m.tzanova@farmasi.uio.no (M.M.T.); 4 Research Institute for Medicines (iMed.ULisboa), Faculty of Pharmacy, Universidade de Lisboa, Avenida Professor Gama Pinto, 1649-038 Lisboa, Portugal; jmmarto@ff.ulisboa.pt (J.M.); hribeiro@campus.ul.pt (H.R.); lgoncalves@ff.ulisboa.pt (L.M.G.); 5 Pharmaceutical Botany Laboratory, Department of Pharmacy and Biotechnology, Alma Mater Studiorum, University of Bologna, Via Irnerio 42, 40127 Bologna, Italy; manuela.mandrone2@unibo.it (M.M.); ilaria.chiocchio2@unibo.it (I.C.)

**Keywords:** *Rosa canina* L. extract, polyphenols, antioxidant activity, ethosomal gel, sodium hyaluronate, anti-aging ingredients, cosmetic products

## Abstract

Rising environmental awareness drives green consumers to purchase sustainable cosmetics based on natural bioactive compounds. The aim of this study was to deliver *Rosa canina* L. extract as a botanical ingredient in an anti-aging gel using an eco-friendly approach. Rosehip extract was first characterized in terms of its antioxidant activity through a DPPH assay and ROS reduction test and then encapsulated in ethosomal vesicles with different percentages of ethanol. All formulations were characterized in terms of size, polydispersity, zeta potential, and entrapment efficiency. Release and skin penetration/permeation data were obtained through *in vitro* studies, and cell viability was assessed using an MTT assay on WS1 fibroblasts. Finally, ethosomes were incorporated in hyaluronic gels (1% or 2% *w*/*v*) to facilitate skin application, and rheological properties were studied. Rosehip extract (1 mg/mL) revealed a high antioxidant activity and was successfully encapsulated in ethosomes containing 30% ethanol, having small sizes (225.4 ± 7.0 nm), low polydispersity (0.26 ± 0.02), and good entrapment efficiency (93.41 ± 5.30%). This formulation incorporated in a hyaluronic gel 1% *w*/*v* showed an optimal pH for skin application (5.6 ± 0.2), good spreadability, and stability over 60 days at 4 °C. Considering sustainable ingredients and eco-friendly manufacturing technology, the ethosomal gel of rosehip extract could be an innovative and green anti-aging skincare product.

## 1. Introduction

Recently, many changes in consumer behaviors have occurred due to the coronavirus pandemic and climate changes [1], and one of them is a rising awareness about the cumulative effects of individual consumption behavior on environmental sustainability [2]. This attitude has been encouraged by the principles of sustainable development adopted by the United Nations, suggesting that all people should strive to meet their needs in a way that preserves, protects, and restores the health and integrity of the Earth’s ecosystem [3]. This rising attention to promoting a more sustainable lifestyle drives green consumers to purchase green products [4]. Hence, consumer attitude towards sustainability is remarkable even in the cosmetic market trends, with rising requests for brands actively involved in ethical or environmentally sustainable practices [5]. Even though the three dimensions of sustainability (social, economic, and environmental) concern the entire cycle of cosmetic production, the choice of raw materials and the product design are the phases with the highest impact on the sustainability of a cosmetic product [6]. For this reason, the selection of botanical ingredients has gained the cosmetics industry’s attention in order to satisfy green consumers’ requests [7,8]. Among botanical ingredients, rosehips could represent a valid green and sustainable raw material for the cosmetics industry. 

Rosehips are the wild edible pseudo-fruits of *Rosa canina* L., a spontaneous shrub resistant to adverse soil and climatic condition, widespread in Asia and Europe due to its high environmental adaptability. Rosehips are a natural source of bioactive compounds known from ancient times for many health benefits, as they have anti-inflammatory, anti-arthritic, analgesic, anti-diabetic, and antimicrobial properties [9]. Rosehips contain many antioxidant compounds such as ascorbic acid and polyphenols. As reported in the literature, among all polyphenols, many phenolic acids have been identified, such as gallic, ellagic, caftaric, chlorogenic, caffeic, coumaric, and ferrulic acids [10,11]. Flavonoid compounds have also been determined, in particular catechins, epicatechin, and quercetin [12,13], suitable for anti-aging cosmetic action [9,14]. Noticeably, the “anti-aging” segment of the cosmetic market is growing parallel to the “natural” segment [7], and rosehip extract could conjugate the preference for a natural source of ingredients with the issue of the rejuvenation of skin.

Polyphenols and ascorbic acid can scavenge reactive oxygen species to reduce oxidative stress and skin damage. These compounds offer a synergistic anti-inflammatory and antioxidant effect. Moreover, ascorbic acid promotes collagen synthesis, resulting in a tightening effect [15]. However, these antioxidant compounds are not stable to the temperature, light, and oxygen action, and their antioxidant activity could be easily lost [16,17]. Based on this limitation, the encapsulation of natural extracts in nanocarriers (liposomes, phytosomes, ethosomes, niosomes, transferosomes) has been demonstrated to be a valid strategy to preserve the antioxidant properties of bioactive compounds and improve their bioavailability [18,19].

Ethosomes are vesicular systems suitable for botanical extract encapsulation and well-studied for skin applications [20,21]. Discovered for the first time by Touitou et al. [22], they are composed of phospholipids, ethanol, and water. Among all the phospholipids, lecithin is a suitable natural option to prepare ethosomes [6]. Ethanol percentage influences the physicochemical characteristics of lipid vesicles, namely size, polydispersion index, zeta potential, and entrapment efficiency. Moreover, ethanol makes ethosomes more flexible, deformable, and elastic than conventional liposomes and, consequently, increases the ability to penetrate through the different skin layers [23]. Ethosomal suspensions could be incorporated in semisolid formulations to improve topical use; hyaluronic acid (HA) was selected as a gelifying natural polymer to obtain an ethosomal gel. 

HA is widely used in cosmetic products to improve skin hydration, elastin, and collagen stimulation. Owing to its tissue regeneration potential, it exhibits anti-wrinkle, anti-aging, and skin rejuvenation properties. Moreover, regarding technological properties, HA is an excellent gelling agent due to its ability to bind water easily [24]. Different concentrations of HA can be used to obtain gels with tunable rheological properties and drug release profiles [25]. Currently, gel formulations are consumers’ most appreciated semisolid dosage forms due to their fast hydration, low stickiness, and light consistency.

This work aimed to encapsulate rosehip extract in lecithin-based ethosomes to improve the stability of bioactive compounds. The ethosomes were characterized through size, polydispersion index, zeta potential, entrapment efficiency, *in vitro* release and skin permeation studies. In addition, a biocompatibility assay was performed on WS1 fibroblasts. Finally, we developed a gel based on hyaluronic acid containing rosehip extract ethosomes; the rheological properties and stability over time were assessed to obtain a green anti-aging cosmetic formulation. 

## 2. Results and Discussion

### 2.1. In Vitro Evaluation of Rosehip Extract Anti-Aging Properties 

The determination of antioxidant activity is an effective approach to ensure the quality of an anti-aging cosmetic formulation. In fact, intracellular and extracellular oxidative stress initiated by reactive oxygen species (ROS) is involved in skin aging.

**Figure 1:**
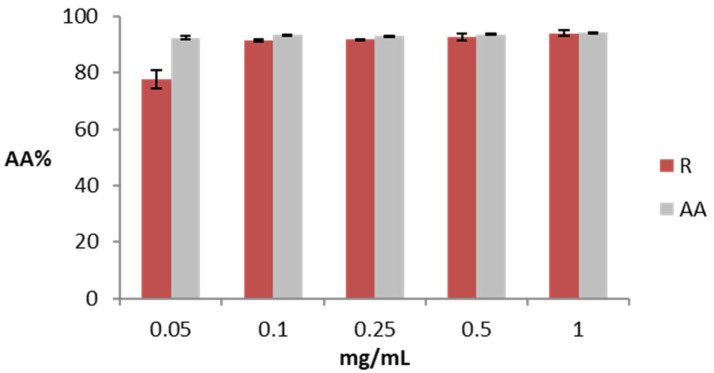
Antioxidant activity of rosehip extract (R) and ascorbic acid (AA). The data are expressed as the mean of three replicate experiments ±SD. Significance: (*) *p* < 0.05.

Figure 1 shows the antioxidant activity measured as the percentage of DPPH reduction for different concentrations of rosehip extract and ascorbic acid (AA) solution, a reference substance for the antioxidant action. Rosehip extract at a low concentration (0.05 mg/mL) has a lower antioxidant activity (77.52%) compared to the same concentration of AA (92.40%). The antioxidant activity of rosehip extract increases at higher concentrations, and at 1 mg/mL, the sample and the control are similar (94.08 and 94.24% for rosehip extract and AA, respectively). 

**Figure 2:**
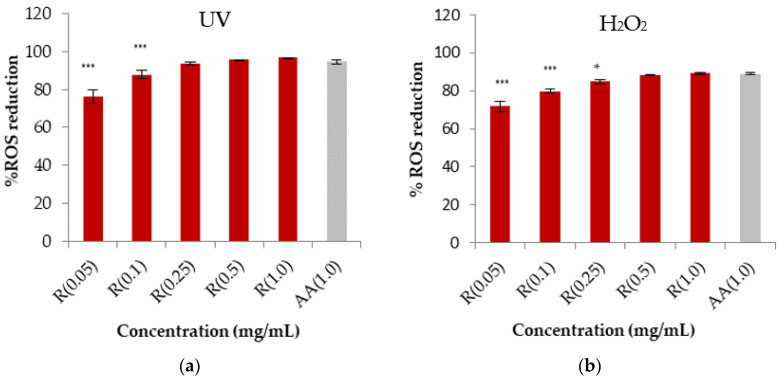
*(***a**,**b**) Reactive oxygen species reduction by rosehip extract solution (R) at different concentrations after exposure to UV and H_2_O_2_ radiation of HaCaT cells. The data are expressed as the mean of at least nine replicate experiments ±SD. Significance: (*) *p* < 0.05 versus positive control cells (ascorbic acid, AA), (***) *p* < 0.001.

In addition, the antioxidant activity was assessed as the capacity to reduce the intracellular generation of ROS induced either by a chemical compound (H_2_O_2_) or by UV light *in vitro* in HaCaT (human keratinocyte cell line). The results are shown in Figure 2a,b.

According to the DPPH assay, a similar trend was found for rosehip extract in the ROS reduction assay, confirming that the highest concentration of rosehip extract is comparable with the control. The IC_50_ was also determined after exposure to H_2_O_2_ and UV radiation, resulting in 37.2 ± 1.1 and 19.4 ± 1.2 µg/mL, respectively. The antioxidant activity of rosehip extract could be due to the extract’s polyphenol and AA content. Based on these results, we selected the concentration of 1 mg/mL of rosehip extract for the continuation of this study as it could be effective in an anti-aging cosmetic product [7,19].

### 2.2. Preparation and Characterization of Ethosomes: Selection of Raw Materials, Influence of Ethanol, and Preparation Technique 

The encapsulation of rosehip extract was performed to protect the antioxidant activity. Specifically, the extract was encapsulated in ethosomes (ET) by using the ethanol injection method. This method was preferred to the thin layer method, the first method used for liposome production, as it is a more green technique. In fact, it avoids pollutant solvents and some evaporation steps, reducing energy consumption, and simple equipment is required to enhance the scalability [26]. 

Concerning the phospholipid for vesicle preparation, lecithin was preferred as a suitable natural option for cosmetic formulations [27].

Finally, different percentages of ethanol ranging from 10 to 40% were tested to select the most promising nanocarrier based on their final physicochemical properties.

These properties can influence the *in vitro* behavior and, consequently, their cosmetic application; for this reason, all types of prepared phospholipid vesicles were characterized in terms of vesicle size (VS), polydispersity index (PDI), ζ potential (mV), pH, and entrapment efficiency (EE%), as reported in Table 1. 

The size of unloaded ethosomes containing 10% of ethanol (ET1) is 312.4 ± 8.5 nm, and this decreases to 178.8 ± 1.5 when the ethanol percentage is 30% (ET3). An increasing percentage of ethanol determines a decrease in the ethosome size due to the interposition of ethanol between the hydrocarbon chains, leading to a reduction in vesicular membrane thickness and, hence, in vesicular size [23,28]. When the percentage of ethanol is 40% (ET4), the size increases to 213.6 ± 3.4 nm, as a high percentage of ethanol leads to more leaky vesicles [29]. According to the literature, intermediate percentages of ethanol lead to ethosomes of minimum sizes; instead, the highest ethanol percentages can lead to the disruption of lipid vesicles [30].

In the case of loaded ethosomes, a similar trend related to the ethanol percentage can be observed. However, the vesicle size of loaded samples increases for all formulations (363.6 ± 7.3 nm, 288.1 ± 8.6 nm, 225.4 ± 7.0 nm, and 240.9 ± 1.9 nm for ET1R, ET2R, ET3R, and ET4R, respectively) compared to unloaded vesicles. This enlargement could be attributed to the interposition of the phytochemicals in vesicle structures. In particular, the more hydrophobic compounds are arranged between the phospholipid bilayer, while the more hydrophilic substances are included in the inner aqueous nucleus, determining the vesicle enlargement [31]. The increase in the size of vesicles determined by polyphenol encapsulation is reported for different natural extracts by other authors [32,33].

The polydispersity index is a parameter to measure the homogeneity of the systems. A value of PDI less than 0.3 indicates that the vesicles have similar dimensions, and the system could be considered homogeneous. Among all prepared formulations, those containing 30% ethanol have the lower value of PDI, resulting in 0.25 ± 0.01 for ET3 and 0.26 ± 0.02 for ET3R, indicating good homogeneity.

Concerning the zeta potential (ζ), this value helps predict the system’s stability. A value of zeta potential of ±30 mV indicates that the vesicles tend to repulse each other and maintain the system stability over time. According to the literature, ethanol acts as a negative charge provider for the surface of ethosomes. All the formulations show negative values of zeta potential less than −40 mV that tend to increase with the ethanol percentage [30,34].

Entrapment efficiency (EE) is a parameter related to the delivery capacity of vesicular systems. Among all types of vesicular systems, ethosomes are characterized by a high value of EE. As reported in Table 1, the EE increases with the increase in ethanol up to 30%. ET3R has the highest value of EE (93.41 ± 5.30%) due to the ethanol effect, meaning that ethanol improves the solubility of hydrophobic and hydrophilic compounds and, consequently, the loading in the vesicles. However, when the ethanol increase is higher, the EE tends to decrease as it could dissolve the membranes [28,30]. 

Finally, it was observed that the pH value ranges from 6.41 ± 0.10 for unloaded ethosomes to 5.44 ± 0.08 for loaded ethosomes. The lower value of loaded ethosomes could be explained by some organic acid in the extract (malic, citric, ascorbic acid). This lowering of pH is favorable for skin application [35].

Based on these results, the ethosomal suspension of ET3R was selected for the study as it had a small size (<300 nm), low PDI (<0.3), and the highest EE (>90%).

### 2.3. Vesicles’ Physical Stability

**Figure 3:**
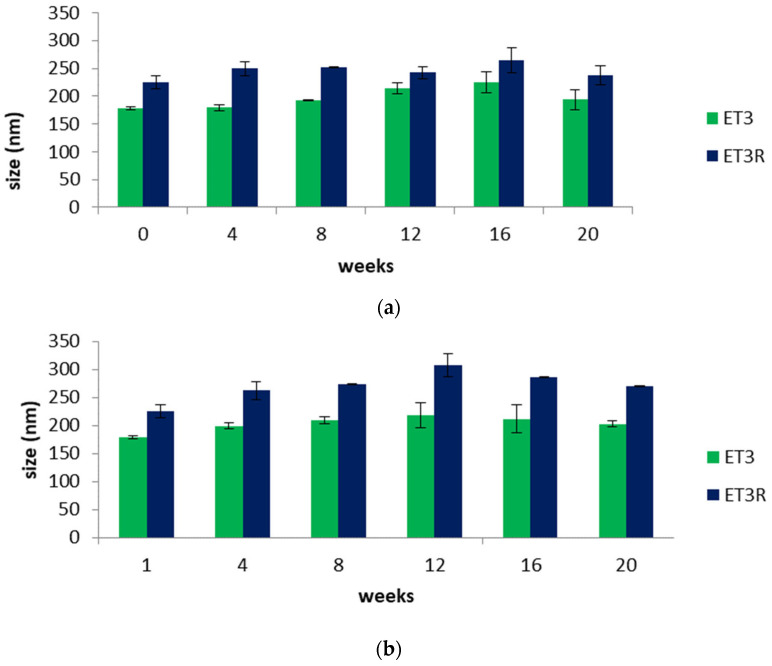
(**a**) Size (nm) of unloaded (ET3) and loaded ethosomes (ET3R) during 20 weeks of storage at 4.0 ± 2.0 °C. Data expressed as mean ± SD (*n* = 3). (**b**) Size of unloaded (ET3) and loaded ethosomes (ET3R) during 20 weeks of storage at 25 ± 2.0 °C. Data expressed as mean ± SD (*n* = 3).

The stability of nanocarriers as ingredients for cosmetic formulations needs to be investigated to guarantee the effectiveness and safety of the skincare product. Figure 3a,b shows the variation in the sizes of unloaded (ET3) and loaded ethosomes (ET3R) over a storage period of 20 weeks at 4.0 and 25 ± 2.0 °C. Unloaded ethosome sizes range from a minimum of 178 nm to a maximum of 225 nm at the lower temperature and a maximum of 217 nm at the higher temperature; these results are in agreement with the literature, confirming that among all the nanocarriers, ethosomes have good stability [21]. Concerning the loaded ethosomes, stability studies show that the enlargement in the dimensions due to the incorporation of extract compounds is maintained over the considered period. Comparing Figure 3a,b, a slight increase in loaded ethosome sizes can be noticed at 25 °C due to the increased fluidity of phospholipid vesicles [30]. However, over the considered period, the size of loaded ethosomes remains less than 300 nm, a size value that is favorable for skin penetration [36,37].

### 2.4. In Vitro Release Studies

The release of polyphenols from ET3R and the rosehip extract solution (1 mg/mL) was performed in a mixture of PBS: EtOH 7:3 *v*/*v* at a temperature of 32 ± 1.0 °C with constant stirring at 100 rpm. The Folin–Ciocalteu test was used to determine the TPC released over a period of 24 h. 

**Figure 4:**
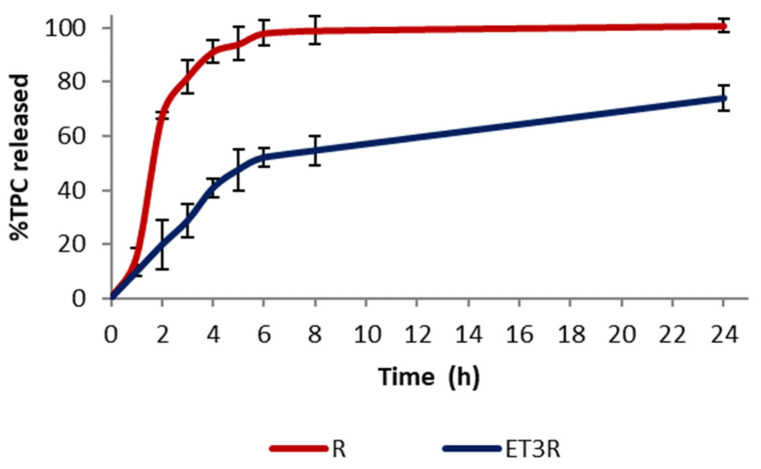
*In vitro* release of TPC in PBS:EtOH (7:3 *v*/*v*) from ET3R and R solution. Data expressed as mean ± SD (*n* = 3).

The *in vitro* release profiles of TPC from the extract solution and from loaded ethosomes ET3R are shown in Figure 4. Rosehip extract exhibited a fast release profile, with 81.67 ± 6.11% of TPC released after 180 min, and reached the maximum release after 24 h. In contrast, the extract encapsulated in phospholipid vesicles showed a slower release over time, with 73.84 ± 4.59% of TPC released after 24 h. This release behavior can favor the delivery of bioactive compounds for cosmetic application [21].

### 2.5. In Vitro Permeation Studies Using an Artificial Biomimetic Barrier

The evaluation of skin absorption is essential for cosmetic efficacy and cosmetic safety. Skin absorption includes penetration (the mass of the test substance that enters the skin) and permeation (the mass transferred from the skin to the reservoir compartment fluid) [38]. An *in vitro* permeation test using a 96-well Permeapad^®^ plate was performed to clarify the different aspects of the absorption of bioactive compounds encapsulated in nanocarriers through the different layers of the skin. The permeability studies using this membrane compared the rosehip extract solution and the rosehip extract loaded into the ethosomes. 

**Figure 5:**
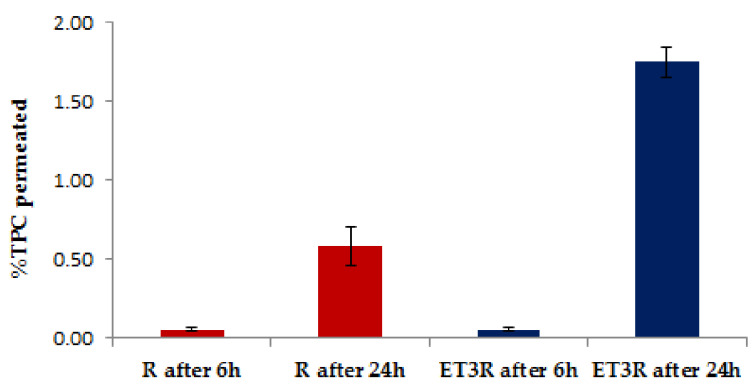
*In vitro* permeation of polyphenols from R solution (1 mg/mL) and ET3R after 6 and 24 h. Data expressed as mean ± SD (*n* = 3).

After 6 h, the amount of TPC was almost undetectable through the Permeapad^®^ 96-well plate (Figure 5). After 24 h, a small amount of TPC (˂2%) for the encapsulated extract was detectable in the receptor compartment, confirming that the encapsulation in ethosomes contributes to the increase in the permeation of bioactive compounds. Further investigations are required to determine the type of polyphenols that pass through the Permeapad^®^ in the receptor compartment after 24 h. A standard Permeapad^®^ has been developed for quantifying the GI and mucosal drug absorption *in vitro*. The Permeapad^®^ technology has been used as a starting point for manufacturing a new skin-mimicking barrier (SMB) suitable for transdermal formulation [38]. However, as this is not a commercial product yet, we focused on the classical 96-well plate PermeaPad^®^. We regarded this approach as acceptable for the scope of this work as the goal of this permeation study was to verify if polyphenols could be potentially transported through a biological barrier. The prepared formulation seems to be suitable for cosmetic use as its systemic absorption should be quite limited, according to the *in vitro* permeability testing results within the application time. 

### 2.6. In Vitro Skin Permeation/Retention Studies Using Pig Ear Skin

**Figure 6:**
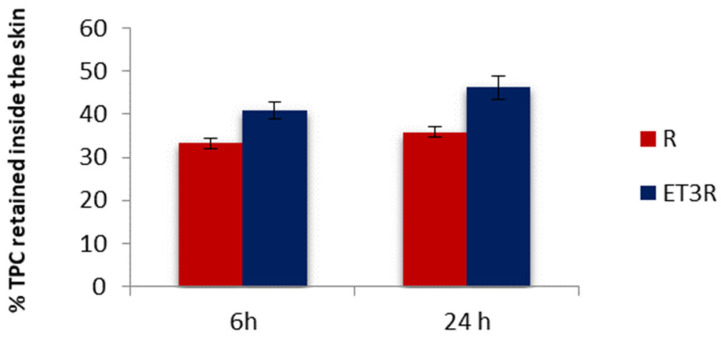
*In vitro* skin retention of TPC from R solution and ET3R after 6 and 24 h. Data expressed as mean ± SD (*n* = 6).

The retention of polyphenols inside the skin layers was assessed using porcine ear skin due to its similarity to human skin. In Figure 6, we compare the percentage of TPC from loaded ethosomes or rosehip extract solution detectable in the porcine skin after 6 and 24 h of permeation.

The TPC percentage amount from ET3R retained inside the skin after 6 and 24 h was 40.99 ± 1.95% and 46.21 ± 2.75%, respectively. For the rosehip extract solution used as control, the TPC retained inside the skin after 6 and 24 h was 33.18 ± 1.21% and 40.92 ± 1.17%, respectively. These results suggested that after 6 h, ET3R increased the retention of TPC in comparison to the control (*p* < 0.05). 

The higher amount of TPC found in the skin for loaded ethosomes could be due to the ethosomes’ properties as penetration enhancers. As reported in the literature, this is the result of two combined effects: (i) the “ethanol effect”, which increases the fluidity of phospholipids in the stratum corneum and (ii) the “ethosomes effect”, which promotes the fusion of vesicles with skin lipids, favoring the penetration into the deeper layers of the skin [30]. Moreover, ethosomes’ lecithin assures higher hydration to the skin compared to the free extract solution, which can promote skin penetration [39]. TPC was not detected in the receptor compartment after 6 h for all samples, confirming the data obtained with the Permeapad^®^ membrane. These data confirm that the prepared formulations are suitable for cosmetic use as the bioactive compounds are intended to have a predominantly local effect [40]. In particular, the concentration of polyphenols in the epidermis and dermis is favorable for the cosmetic action of a skincare product [41]. 

### 2.7. In Vitro Cytotoxicity Assay

The biocompatibility of the ethosome suspensions was tested through an MTT assay on WS1 fibroblasts, the primary cells of connective tissue, producing collagen and elastin, and the target of the topical formulations. 

**Figure 7:**
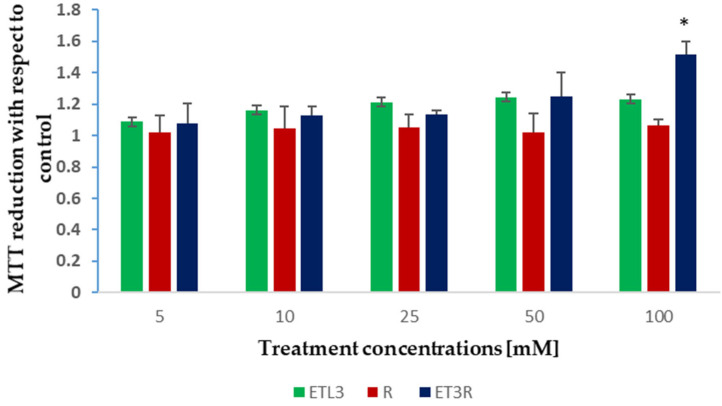
MTT assays on WS1 cells treated for 24 h with increasing concentrations of ET3, R solution, and ET3R. Effects on cell viability were normalized in comparison with control cells and analyzed using one-way ANOVA (* *p* < 0.05).

WS1 fibroblasts were treated for 24 h with the rosehip extract solution, or unloaded or loaded ethosomes, in a range of concentrations between 5 and 100 µg/mL. The data showed that none of the treatments had a detrimental effect on WS1 cells (Figure 7). However, at the highest concentration, only the rosehip extract encapsulated in the ethosomes produced a significative increment in cell viability, as indicated in the increased absorbance of metabolized formazan salt in these samples. In similar studies, other authors reported that ethosomes are safe and present excellent skin tolerability [21].

### 2.8. Development of Rosehip Ethosomal Gel

#### 2.8.1. Physicochemical Characterization of Rosehip Ethosomal Gels

Gels are currently the preferred formulations for anti-aging products and the most suitable semisolid formulation to incorporate phospholipid vesicles [42]. Compared to liquid suspensions, hydrogel formulations increase consistency and adhesiveness and facilitate skin application. For this reason, ethosomal suspensions were integrated into low-molecular weight hyaluronic gels. Among natural gelling polymers, hyaluronic acid was selected for its moisturizing effect and anti-aging properties. HA percentages of 1 and 2% were selected to increase the viscosity and facilitate skin application, considering that the usual percentage of HA in cosmetic products ranges from 0.2 to 2% [43]. The viscosity (cP), pH value, spreadability (mm^2^), and particle size of ethosomal gels were measured at room temperature 48 h after their preparation. 

In Table 2, the range of viscosity values is between 305.0 ± 7.0 cP and 403.3 ± 11.5 cP for formulations based on HA1% and between 3166.7 ± 57.7 cP and 3233.3 ± 155.5 cP for those at HA2%. Concerning the effects of the different compounds in the formulations, a higher concentration of the polymer HA increases the viscosity. In the HA1% formulations, the presence of rosehip extract leads to a decrease in viscosity. In contrast, lecithin ethosomes lead to an increase in viscosity in all formulations. A similar result was reported by Ramadon et al. [34]. Concerning the HA2% formulations, viscosity values increase owing to the higher polymer concentration.

Regarding pH, formulations containing rosehip extract have a lower pH value, between 5.5 and 5.6, due to some organic acids in the extract (i.e., malic, citric, ascorbic acid). This lowering of pH makes the formulations suitable for skin application [35].

Spreadability is one of the most important characteristics of a semisolid formulation. It is essential to ensure that bioactive substances are distributed well and quickly for their topical delivery at the moment of skin application, increasing consumer compliance [44]. The spreadability of the prepared gels is reported in Table 2. A decrease in spreadability was observed with the increase in viscosity, and the ethosomal gel containing HA1% was more spreadable than the HA2% gel.

Finally, regarding the size of vesicles, an increase in this value can be observed compared to the ethosomal suspensions, which could be due to the wrapping of HA on the surface of vesicles [45].

#### 2.8.2. Rheological Properties of Ethosomal Gels

Rheological measurements are essential to developing a final formulation acceptable for consumers as they give preliminary information about spreading, adhesiveness, or lubrication in skin application. 

**Figure 8:**
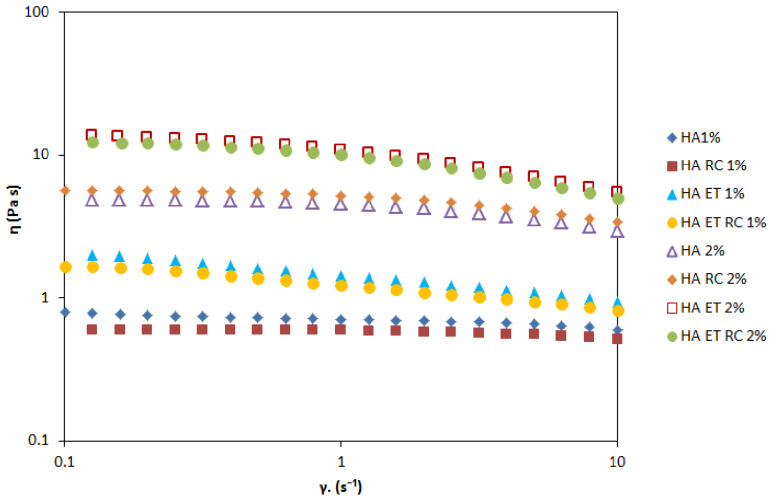
Viscosity as a function of shear rate for ethosomal gels. These curves describe the flow behavior of the sheared and time-independent fluid.

**Figure 9:**
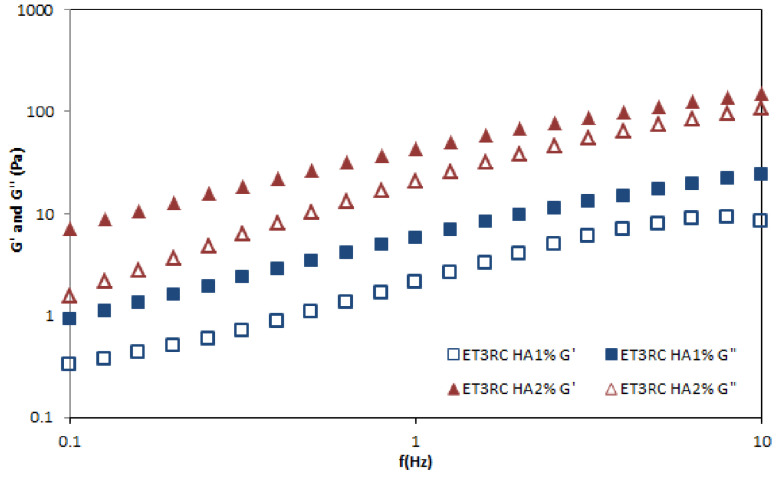
Frequency sweep with shear moduli as a function of the frequency of loaded hyaluronic gels ET3R HA1% and ET3R HA2% at room temperature.

Figure 8 shows the profile of all prepared gels in terms of the shear rates. The results suggested that gel viscosity increases with the percentage of HA. However, each gel can be classified as a shear-thinning fluid (non-Newtonian system) because the viscosity decreases as the shear rate increases. An oscillation frequency sweep test was performed to assess the viscoelastic behavior of the prepared formulations. This test could provide more information about the effect of colloidal forces and interactions among particles [46]. A preliminary amplitude sweep test defined the fluid’s LVER, and the results showed that this region was at 2% shear strain. Figure 9 shows the frequency behavior of HA1% and 2% loaded gels. The higher concentration of HA increases the value of the elastic modulus G’ and viscous modulus G”. At all the measured frequencies, the G” modulus is higher than G’ modulus, indicating that both formulations exhibited fluid-like behavior.

The adhesiveness test of gel formulations measures the force required to separate the product from the test surface. The higher the force to separate these surfaces, the more adhesive the cosmetic product is. Table 3 shows the results of the adhesion test for all prepared gels. 

The data showed that the formulation ET HA2% has the highest adhesiveness, with a peak normal force of −1.436 ± 0.056 N and an area under force–time curve of 1.567 ± 0.192 N·s. Similarly to the viscosity data, the increase in hyaluronic acid concentration and lecithin’s presence in ethosomes increase the adhesiveness. In contrast, the presence of the extract decreases the adhesiveness when it is non-encapsulated [47].

### 2.9. In Vitro Release from Gels

The *in vitro* release studies were performed for gel formulations following the same method applied for ethosomal suspensions to investigate the polyphenols’ release.

**Figure 10:**
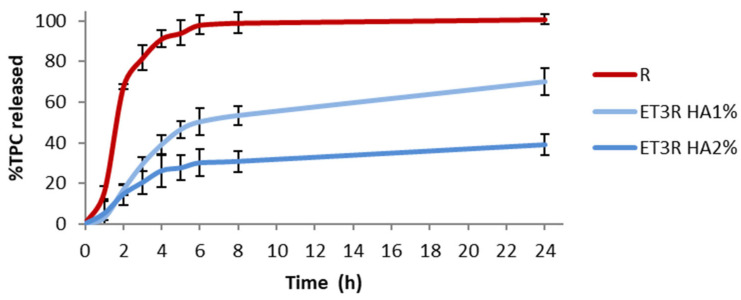
*In vitro* release of TPC in PBS:EtOH (7:3 *v*/*v*) from R solution (1 mg/mL) and loaded ethosomal gels (HA1% and HA2%). Data expressed as mean ± SD (*n* = 3).

Figure 10 shows the release profiles of the prepared gel formulations. The TPC released after 6 h is 50.19 ± 6.60% for HA1% and 30.10 ± 6.72% for HA2%.

The HA2% gel formulation showed a slower release than HA1%, which could be due to the higher viscosity slowing the diffusion of bioactive compounds. Other studies reported that the inclusion of ethosomes in the gel can give an additional modulation in the retention time [20,21]. These results confirmed that HA as the gelling agent could modulate the release of polyphenols, depending on concentration [24]. 

On the other hand, the release profile from the ET3R HA1% formulation seems more suitable for a skincare anti-aging product, and we selected this formulation to assess its stability over time.

### 2.10. Stability Studies of Gels

The stability test is fundamental to establishing the shelf life of a cosmetic product and checking that the effects of the environmental conditions do not change its quality and, consequently, its skin benefits. The stability studies for the most suitable gel formulation were carried out for 60 days to assess any change in organoleptic characteristics, viscosity, pH, and vesicle size at 4° and 25 ± 2.0 °C at specific time intervals. The results are shown in Table 4. 

Regarding the organoleptic characteristics, the aspect, color, and odor of gels stored at 4.0 ± 2.0 °C remained similar over the period considered. However, for the gel stored in standard conditions, some changes were observed, namely an increase in the vesicle size of the loaded gel and a color change from cream gold (Pantone 13-0739TCX) to old gold (Pantone 15-0955TCX), likely due to the oxidation process at this temperature; similar color changes in ethosomal gels were reported by Ramadon et al. [34]. Based on the viscosity measurement, an increase over time in refrigerated conditions and an initial decrease in standard conditions were observed, suggesting a direct correlation with the temperature. This increase is higher for gels containing ethosomes, likely due to the more significant effect of temperature on lecithin’s physical state. For the loaded gels, the viscosity ranged from a minimum of 397.5 ± 17.1 cP to a maximum of 440.0 ± 28.3 cP at 4.0 ± 2.0 °C. This minimal increase indicates the substantial physical stability of the gel formulation concerning the viscosity up to 60 days at refrigerated temperatures. The pH of loaded formulations remains in the range of 5.1–5.7, indicating that the prepared gel remains suitable for skin application over time. 

## 3. Conclusions

An ethosomal gel of rosehip extract was successfully prepared using hyaluronic acid as a gelling agent. *In vitro* anti-aging properties of rosehip extract were tested through a DPPH assay and ROS reduction test; rosehip extract exhibited antioxidant activity similar to a solution of ascorbic acid. Rosehip extract was encapsulated in lecithin ethosomes and the ethosomal suspensions, and ET3R containing 30% ethanol was selected for its optimal characteristics: small size, low polydispersity, and good entrapment efficiency. The *in vitro* release studies showed that encapsulation leads to a delay in the release of the extract. The permeation and retention studies through several membranes reveal that the amount of TPC in the skin from the encapsulated extract was higher than that from the un-encapsulated extract. Moreover, TPC was not detectable in the receptor compartment after 6 h, confirming the appropriate cosmetic use of the formulation. Finally, hyaluronic acid 1 or 2% (*w/v*) was added to the ethosomal suspension to improve the anti-aging action and facilitate skin application. Ethosomal gel of 1% (*w/v*) hyaluronic acid is a promising cosmetic formulation with a suitable pH for skin use, easy spreadability, and stability regarding viscosity and pH, and it could be considered a green, sustainable, and effective anti-aging cosmetic as it is made with sustainable ingredients, an easily scalable production process, and well-known anti-aging bioactive compounds.

## 4. Materials and Methods

### 4.1. Materials

The hydroalcoholic extract was obtained from lyophilized rosehips [48]. Methanol and ethanol were purchased from Sigma-Aldrich (Milan, Italy). Sodium hyaluronate (molecular weight = 800–1200 kDa) was sourced from Farmalabor (Canosa di Puglia, Italy). Folin–Ciocalteu reagent was sourced from Titolchimica (Pontecchio Polesine, Italy). Soy lecithin and other chemicals were purchased from Carlo Erba (Milan, Italy). Sodium phosphate dibasic anhydrous, sodium phosphate monobasic anhydrous, and Dulbecco’s modified Eagle medium supplemented with D-glucose were purchased from Sigma-Aldrich Co. (St. Louis, MO, USA). Fetal bovine serum (FBS), L-glutamine, penicillin (1000 U/mL), and streptomycin were purchased from Euroclone S.p.A. (Milan, Italy).

### 4.2. In Vitro Evaluation of Rosehip Extract Anti-Aging Properties 

#### 4.2.1. Antioxidant Activity of Rosehip Extract according to DPPH Method

The antioxidant activity of rosehip extract was determined using the 2,2-diphenyl-1-picrylhydrazyl radical (DPPH, Sigma Aldrich, Milan, Italy) reduction assay as described by Brand-Williams et al. [49] with minor modification. Briefly, 1 mL of rosehip extract solution at different concentrations ranging from 0.05 to 1 mg/mL was added to 1 mL of DPPH methanol solution (0.1 mM). The mixture was stored in the dark for 30 min, then the absorbance was spectrophotometrically measured at 517 nm. Methanol:water 50:50 *v*/*v* was used as a blank solution and the DPPH solution as the control. The test was carried out in triplicate. The results are expressed as a percentage of inhibition of the DPPH radical according to the following equation: inhibition % = [(A_0_ − A)/A_0_], where A_0_ is the absorbance of the DPPH control and A is the absorbance of the sample.

#### 4.2.2. Antioxidant Activity of Rosehip Extract according to Reactive Oxygen Species (ROS) Production Measurement

The intracellular production of ROS within cells was assessed with a fluorimetric technique using 2′,7′-dichlorodihydrofluorescein diacetate (H2-DCFDA, Life Technologies, Paisley, UK) [50]. HaCaT (human keratinocyte cell line, CLS, Eppelheim, Germany) sub-confluent cells grown in 96-well plates were incubated for 30 min with 20 μM of H2-DCFDA in the dark at 37 °C. The media was removed and fresh medium was added to the cells before they were exposed to different concentrations of samples (0.05–1 mg/mL) and ascorbic acid (AA, 1 mg/mL) for 1.5 h. Hydrogen peroxide (H_2_O_2_) solution (500 μM, 1 h) or exposure to UVB light (emission wavelength 312 nm) for 15 min were used for the induction of ROS in cells. After exposure, ROS levels were determined at excitation 485 nm and emission 520 nm wavelengths using a fluorescence microplate reader (FLUOstar BMGLabtech, Offenburg, Germany). Data from 9 replicates are reported as the percentage of ROS reduction determined as 100-(fluorescence of exposed cells/fluorescence of unexposed control from the same experiment) × 100. The concentration of the samples that reduced 50% of ROS (IC_50_) was determined through non-linear regression analysis with GraphPad PRISM^®^ 5 software (San Diego, CA, USA, www.graphpad.com, (accessed on 16 November 2022).

### 4.3. Preparation of Ethosomes: Selection of Raw Materials, Influence of Ethanol, and Preparation Technique 

Ethosomes were prepared through the ethanol injection–sonication method reported by Ma et al. [51] with minor modifications. Briefly, soy lecithin (100 mg) was dissolved in different volumes of ethanol, ranging from 1 to 4 mL, in a covered beaker to avoid ethanol evaporation. The rosehip extract (10 mg) was dissolved in double-distilled water and mixed uniformly with a magnetic stirrer. The ethanolic solution of phospholipids was slowly added to the aqueous solution of rosehip extract with a syringe at 1 mL/min under constant stirring at 700 rpm to obtain four different formulations, named ET1R, ET2R, ET3R, and ET4R, containing an ethanol percentage of 10, 20, 30, and 40%, respectively. The resulting vesicle suspensions were homogenized through ultrasonication for 15 min (Elma Transonic T310, Singen, Germany) to convert large multilamellar vesicles into smaller vesicles [28]. Unloaded ethosomes (ET1, ET2, ET3, and ET4) were prepared as a control. 

### 4.4. Vesicle Characterization

#### 4.4.1. Size and Zeta Potential Measurements 

The prepared ethosomes were characterized for their vesicle size (VS) and their polydispersity index (PDI). The ethosome suspensions were diluted (1:800 *v*/*v*) in ultrapure water (18.2 MΩ cm, MilliQ apparatus by Millipore, Milford, MA, USA). Size and PDI were measured through PCS (photon-correlation spectroscopy) using a Brookhaven 90-PLUS instrument (Brookhaven Instruments Corp., Holtsville, NY, USA) with a He-Ne laser beam at a wavelength of 532 nm (scattering angle of 90°). The measurements were performed at room temperature with five runs for each determination. The zeta potential measurements were carried out at 25 °C on a Malvern Zetasizer 3000 HS instrument (Malvern Panalytical Ltd., Malvern, UK) after the same dilution. 

#### 4.4.2. Entrapment Efficiency 

The entrapment efficiency (EE) of the ethosomes was determined through the dialysis method. To evaluate the amount of bioactive compounds encapsulated into the vesicles, samples (1 mL) were purified from the non-incorporated components through dialysis (Spectra/Por^®^ membranes: 12–14 kDa MW cut-off) in water (0.5 L) for 2 h at room temperature, with distilled water refreshed after 30 min (2 L in total amount). At the end of the purification process, the antioxidant activity (inhibition %) of the samples before and after dialysis was measured using the DPPH assay, and the EE was calculated as a percentage of the antioxidant activity after dialysis versus that before dialysis as in the following formula: EE% = (inhibition % dialyzed sample/inhibition% non-dialyzed sample) × 100.

#### 4.4.3. pH Measurement 

The pH of various ethosomal suspensions was determined by using a digital pHmeter (Crison Instruments, S.A. Barcelona, Spain). The glass electrode was calibrated with the solutions determined for the equipment (pH of 4.00 and 7.00). 

#### 4.4.4. Physical Stability

The physical stability of the unloaded and loaded ethosomes with 30% ethanol (ET3 and ET3R) was assessed by monitoring the size and the PDI over 20 weeks of storage at 4.0 and 25.0 ± 2.0 °C in the dark. For this study, at predetermined periods (0, 4, 8, 12, 16, and 20 weeks) aliquots of vesicle suspensions were diluted in ultrapure water (1:800; *v*/*v*), and the change in ethosome size and PDI were measured through DLS as reported in Section 4.4.1.

### 4.5. In Vitro Release Studies 

The polyphenol release profiles from ethosomes were investigated using a Franz-type static glass diffusion cell (15 mm jacketed cell with a flat-ground joint, and clear glass with a 12 mL receptor volume; diffusion surface area = 1.77 cm^2^) equipped with a V6A Stirrer (PermeGearInc., Hellertown, PA, USA). A cellulose membrane (MF-Millipore cellulose nitrate 0.22 μm, Sartorius Stedim, Biotech GmbH, Germany) was placed between the receptor and the donor compartments, and 12 mL of a mixture of 3:7 (*v*/*v*) ethanol/pH 7.4 buffer was used as the receptor medium. The donor compartment was filled with 1 mL of vesicle suspension. The systems were kept at 32.0 ± 1.0 °C under magnetic stirring (100 rpm/min). Aliquots (0.2 mL) were withdrawn at predetermined intervals, and the release medium was refilled with the same volume. The polyphenols were determined through UV-Vis spectrophotometry using the Folin–Ciocalteu method, and the amount released from ethosomes was compared with the simple dissolution of the extract in the same medium. The polyphenol release profile test was performed in triplicate for each formulation.

### 4.6. In Vitro Permeation Studies Using an Artificial Biomimetic Barrier

Rosehip polyphenol permeation through an artificial biomimetic barrier was investigated using a high-through put 96-well Permeapad^®^ plate (InnoMe GmbH, Espelkamp, Germany) [52]. The Permeapad^®^ represents the first new approach to investigating drug permeability using a biomimetic artificial barrier. It consists of two regenerated cellulose membranes enclosing a layer of dry phospholipids between them, having a thickness of 0.10 mm. Once hydrated, this barrier forms a liposomal gel that, in structure and composition, reassembles a cellular monolayer and accounts for paracellular drug transport [53]. A mixture of PBS pH 7.4/Ethanol 7:3 (*v*/*v*) was used as the acceptor solution (400 µL). The donor (200 µL) compartments were filled using rosehip extract solution (1 mg/mL) and loaded and unloaded ethosomal suspensions. After filling both the acceptor and donor compartments, the plates were incubated in an orbital shaker–incubator (ES-20, Biosan, Riga, Latvia, LV) at 25 °C and 200 rpm for 6 and 24 h, respectively. Samples (100 µL) were taken from the acceptor every 60 min and the withdrawn volume was replaced with fresh release medium. For polyphenol quantification, the samples were directly transferred upon withdrawal to a UV-transparent 96-well microliter plate (Corning Inc., Kennebunk, ME, USA), and the absorbance was measured at 280 nm on a microplate spectrophotometer (SpectraMax 190, Molecular Devices Inc., Sunnyvale, CA, USA). Standard solutions (concentration range: 0.01–0.5 mg/mL) were measured on the same plate, and blank absorption (PBS/Ethanol 7:3 (*v*/*v*) or unloaded ethosomes) was deducted from all measurements’ UV-Vis spectra. Three replicates were assessed for each sample. The percentage of polyphenols permeated was determined using the calibration curve of rosehip extract (R = 0.9966).

### 4.7. In Vitro Skin Permeation/Retention Studies Using Pig Ear Skin

*In vitro* skin permeation and retention studies were performed on Franz diffusion cells using porcine ear skin as a membrane model. Porcine ears were provided by a local slaughterhouse (CLAI, Faenza, Italy), and full-thickness skin was isolated using a method previously reported by Makuch et al. [54]. The excised circular pig skins with 1.60 mm thickness were placed between the donor and receptor compartments, with the inner part of the skin facing the upper inside portion of the cell. Ethosomal suspensions and extract solution (1 mL) were loaded on the skin in the donor compartment. The receptor medium consisted of a 3:7 (*v*/*v*) ethanol/buffer pH 7.4 solution and it was maintained under magnetic stirring at a temperature of 32.0 ± 1.0 °C throughout the experiment. For the permeation studies, aliquots (200 mL) were withdrawn after 1, 2, 4, 6, and 24 h from the receptor medium and immediately replaced with an equal volume of fresh medium. The Folin–Ciocalteu test was used to detect the presence of polyphenols in withdrawn aliquots. At the end of the 6 or 24 h permeation study, the skin was removed from the Franz cells and the excess formulation was gently removed using a cotton swab. Then, the skin was cut into tiny pieces, placed into separated beakers, and extracted with 5 mL of a mixture of ethanol/water 50:50 (*v*/*v*). Beakers were covered to avoid the evaporation of the solvent and stirred at 300 rpm for 24 h at room temperature. The samples were centrifuged and spectrophotometrically analyzed by using the Folin–Ciocalteu method to determine the amount of TPC retained in the skin. Six replicates were performed for each experiment.

### 4.8. Cell Viability Studies

WS1 cells (American Type Culture Collection, ATCC, Manassas, VA, USA) were used, and were grown routinely in 5% CO_2_/humidified air at 37 °C with Dulbecco’s modified Eagle medium supplemented with D-glucose (4.5 g/L), FBS (10% *v*/*v*), L-glutamine (2 mM), penicillin (1000 U/mL), and streptomycin (1 mg/mL). Cells were seeded at 5.000 cells/well (15 × 10^3^ cell/cm^2^) in a 96-well plate (Corning^®^, Corning, NY, USA) and incubated for 24 h to allow cell adhesion. After adhesion, the medium was removed and replaced with fresh medium containing the desired concentration of the compounds to be tested. To test the cell viability, an MTT assay was performed according to Calonghi et al. [55]. Briefly, after 24 h of treatment, 0.01 mL of MTT dissolved in DPBS at a concentration of 5 mg/mL was added to each well, and they were incubated for 4 h at 37 °C in the dark. Then, the medium was removed, and the reduced formazan crystals were dissolved in 0.1 mL pure isopropanol and the absorbance at 570 nm was measured using a multiwell plate reader (Tecan, Männedorf, CH). Data were analyzed using Prism GraphPad software (San Diego, CA, USA, www.graphpad.com, (accessed on 16 November 2022)).

### 4.9. Preparation of Ethosomal Gels

To prepare the ethosomal gels, sodium hyaluronate (HA 1 or HA 2% *w*/*v*) was added to 20 mL of vesicle suspension as a gelling agent, and the suspensions were stirred at 200 rpm for 24 h at room temperature to obtain a homogenous gel.

### 4.10. Physicochemical Characterization of Ethosomal Gels

#### 4.10.1. Measurement of pH, Viscosity, and Spreadability

All gels were characterized in terms of pH, viscosity, and spreadability.

The pH of the gels was determined by using a digital pHmeter (Crison Instruments, S.A. Barcelona, Spain). 

The viscosity was measured using a rotational viscometer Multi-Visc Rheometer (Fungilab, Barcelona, Spain) at 4 and 25 ± 2 °C. After thermal equilibration, the hydrogels were sheared using standard spindles (TR8 and TR10 for gels containing HA 1% and HA 2%, respectively) at a maximum rotational speed of 100 rpm. Viscosity values were expressed in cP.

A parallel-plate method was adopted to measure the spreadability [44]. Gel samples (300 μL) were spread between two Sil-Tec medical-grade solid silicone sheets of rubber 304 mm × 304 mm × 0.127 mm (Technical Products Inc., Lawrenceville, GA, USA). A circle of 20 cm diameter and 200 g weight was applied to them for 1 min. The change in diameter was measured. Measurements were performed at room temperature (25 ± 2 °C). Spreadability in mm^2^ was calculated by the formula: *S* = *d*^2^ × *π*/4, where *S* is the spreading area (mm^2^) and d is the spreading area diameter (mm).

Particle size measurements were performed after dilution in ultrapure water following the procedure described in Section 4.4.1.

All measurements were performed in triplicate.

#### 4.10.2. Rheological Measurements

The rheological studies were conducted on HA gels that had been stored at room temperature for 2 days after their manufacture. The investigations were performed with a controlled stress Malvern Kinexus Rheometer Lab+ (Malvern Instruments, Worcestershire, UK) using a plate and plate geometry. 

Viscosity measurements were carried out between 0.1 and 10 s^−1^. All measurements were performed at 25.0 ± 0.2 °C. The viscosity values (Pa·s) were reported as a function of the shear rate. 

The viscoelastic properties of the produced fluid gels were measured through small amplitude oscillatory experiments at a fixed strain of 2%, which was within the linear region, using a frequency range between 0.1 and 10 Hz. Measurements were performed at 25.0 ± 0.2 °C.

The tackiness and adhesiveness of gels were measured using the same equipment with a plate and plate geometry (pull-away assay or tackiness testing). A toolkit with the conditions of 0.1 mm/s, 5 mm, and 0.15 gap was selected. In this test, the peak of negative normal force required to separate the two parallel plates holding the gel can be attributed to tack. The area under the force–time curve represents the adhesive strength. Six measurements were performed for each sample.

### 4.11. In Vitro Release Studies from Ethosomal Gels

The polyphenol release profiles from HA gels were investigated using a Franz-type static glass diffusion cell. Briefly, 0.5 mL of HA gels, unloaded and loaded with rosehip extract, was placed in the donor compartment, and the release studies were performed as reported in Section 4.5.

### 4.12. Stability Studies of Gels

Stability studies were conducted by keeping the control and the tests of the selected formulations in refrigerated conditions (4 ± 2 °C) and standard conditions (25 ± 2 °C) for 60 days. The formulations were evaluated on the initial day and after 7, 15, 30, and 60 days according to their pH, viscosity, and organoleptic properties checked by visual inspection. The stability studies were performed in triplicate for each type of gel formulation.

### 4.13. Statistical Analysis

The experiments were performed in triplicate and the results are expressed as mean ± standard deviation (SD). ANOVA was used to determine statistical significance. Differences were deemed significant for *p* < 0.05. 

## Figures and Tables

**Table 1:** VS (nm), PDI, ζ potential (mV), EE (%), and pH of ethosomes unloaded and loaded with rosehip extract.

Samples *	Size (nm)	PDI	ζ (mV)	EE%	pH
ET1	312.4 ± 8.5	0.30 ± 0.02	−52.12 ± 1.33	-	6.41 ± 0.10
ET2	223.8 ± 5.4	0.25 ± 0.02	−53.40 ± 1.70	-	6.30 ± 0.01
ET3	178.8 ± 1.5	0.25 ± 0.01	−53.12 ± 1.26	-	6.22 ± 0.04
ET4	213.6 ±3.4	0.25 ± 0.01	−54.13 ± 0.87	-	6.15 ± 0.05
ET1R	363.6 ± 7.3	0.35 ± 0.03	−49.57 ± 1.48	68.78 ± 5.08	5.44 ± 0.08
ET2R	288.1 ± 8.6	0.30 ± 0.10	−51.42 ± 0.95	89.42 ± 2.21	5.47 ± 0.09
ET3R	225.4 ± 7.0	0.26 ± 0.02	−52.99 ± 1.40	93.41 ± 5.30	5.53 ± 0.05
ET4R	240.9 ± 1.9	0.29 ± 0.05	−53.19 ± 1.08	87.10 ± 3.94	5.61 ± 0.06

* ET1, ET2, ET3, and ET4: ethosomes containing an ethanol percentage of 10, 20, 30, and 40%, respectively. ET1R, ET2R, ET3R, and ET4R: ethosomes containing rosehip extract and an ethanol percentage of 10, 20, 30, and 40%, respectively. Data expressed as mean ± SD (*n* =3).

**Table 2:** Viscosity (cP), pH value, spreadability, and particle size of ethosomal gels.

Sample	Viscosity (cP) *	pH *	Spreadability (mm^2^) *	Size (nm) *
HA1%	305.0 ± 7.0	6.3 ± 0.1	4435.58 ± 90.28	
R HA1%	250.0 ± 1.0	5.6 ± 0.2	5025.57 ± 218.91	-
ET3 HA1%	403.3 ± 11.5	6.1 ± 0.1	3995.13 ± 128.71	281.5 ± 2.1
ET3R HA1%	397.5 ± 17.1	5.6 ± 0.2	3998.79 ± 319.52	363.5 ± 1.8
HA 2%	3233.3 ± 55.5	6.8 ± 0.2	2404.72 ± 133.28	-
R HA2%	3166.7 ± 57.7	5.6 ± 0.2	2178.11 ± 96.08	-
ET3 HA2%	3233.3 ± 57.7	7.0 ± 0.2	1556.20 ± 126.94	219.4 ± 10.4
ET3R HA2%	3233.3 ± 57.5	5.5 ± 0.2	1566.33 ± 40.34	289.7 ± 6.4

* Values are expressed as mean ± SD, (*n* = 3).

**Table 3:** Normal force and area under force–time curve results for ethosomal gels.

Formulation	Peak Normal Force–Normal Force (N) *	Area Under Force–Time Curve (N·s) *
HA1%	−0.244 ± 0.020	0.514 ± 0.047
R HA1%	−0.257 ± 0.012	0.502 ± 0.049
ET3 HA1%	−0.399 ± 0.026	0.721 ± 0.056
ET3R HA1%	−0.329 ± 0.015	0.743 ± 0.093
HA 2%	−1.031 ± 0.039	1.294 ± 0.129
R HA2%	−1.039 ± 0.021	1.161 ± 0.143
ET HA2%	−1.436 ± 0.056	1.567 ± 0.192
ET3R HA2%	−1.333 ± 0.036	1.385 ± 0.043

* Values are expressed as mean ± SD, (*n* = 6).

**Table 4:** Stability of ethosomal gels at different storage conditions.

Gel Formulation *	Temperature (°C)	Time (Day)	Viscosity (cP)	pH	Vesicle Size (nm)
ET3 HA1%	4 ± 2 °C	1	403.3 ± 11.5	6.1 ± 0.1	281.5 ± 2.1
		7	405.5 ± 21.2	6.1 ± 0.1	259.5 ± 12.0
		15	415.0 ± 7.1	6.0 ± 0.1	284.5 ± 1.1
		30	426.7 ± 20.8	6.0 ± 0.1	304.0 ± 8.1
		60	415.0 ± 21.2	5.9 ± 0.1	283.4 ± 5.8
ET3 HA1%	25 ± 2 °C	1	403.3 ± 11.5	6.1 ± 0.1	281.5± 8.1
		7	350.5 ± 10.2	6.1 ± 0.1	276.0 ± 18.4
		15	375.1 ± 21.2	6.1 ± 0.1	282.2 ± 12.5
		30	410.5 ± 14.4	5.9 ± 0.1	363.7 ± 18.6
		60	420.0 ± 56.6	5.9 ± 0.1	355.2 ± 8.5
ET3R HA1%	4 ± 2 °C	1	397.5 ± 17.1	5.6 ± 0.2	363.5 ± 1.8
		7	440.3 ± 14.1	5.4 ± 0.2	352.5 ± 19.2
		15	445.5 ± 7.1	5.4 ± 0.2	371.0 ± 19.8
		30	435.0 ± 21.2	5.7 ± 0.2	405.9 ± 17.8
		60	440.0 ± 28.3	5.5 ± 0.2	382.7 ± 9.8
ET3R HA1%	25 ± 2 °C	1	397.5 ± 17.1	5.6 ± 0.2	363.5 ± 1.8
		7	375.5 ± 7.1	5.4 ± 0.2	380.5 ± 10.6
		15	410.0 ± 28.3	5.2 ± 0.2	391.0 ± 46.7
		30	425.0 ± 21.2	5.4 ± 0.2	418.1 ± 7.7
		60	400.0 ± 28.3	5.4 ± 0.2	457.4 ± 8.9

* ET3 HA1% (unloaded ethosomes in hyaluronic acid gel 1%), ET3R HA1% (loaded ethosomes in hyaluronic acid gel 1%). Values are expressed as mean ± SD, (*n* = 3).

## Data Availability

The data are contained within the article.
